# Ageism during the 2024 U.S. Presidential Election: thematic analysis of tweets

**DOI:** 10.1093/geront/gnaf166

**Published:** 2025-07-26

**Authors:** Juanita-Dawne R Bacsu, Ali Akbar Jamali, Alixe Ménard, Dylan Fiske, Megan E O’Connell, Megan Funk, Shirin Vellani, Melba Sheila D’Souza, Florriann Fehr, Jasmine C Mah, Sarah Anne Fraser, Alison L Chasteen, Melissa K Andrew, Shoshana Green, Sepideh Mansourigovari, Raymond J Spiteri

**Affiliations:** School of Nursing, Thompson Rivers University, Kamloops, British Columbia, Canada; Department of Computer Science, University of Saskatchewan, Saskatoon, Saskatchewan, Canada; Interdisciplinary School of Health Sciences, University of Ottawa, Ottawa, Ontario, Canada; Department of Science, Thompson Rivers University, Kamloops, British Columbia, Canada; Department of Psychology & Health Studies, University of Saskatchewan, Saskatoon, Saskatchewan, Canada; School of Nursing, Thompson Rivers University, Kamloops, British Columbia, Canada; Toronto Rehabilitation Institute, University Health Network (UHN), Toronto, Ontario, Canada; School of Nursing, Thompson Rivers University, Kamloops, British Columbia, Canada; School of Nursing, Thompson Rivers University, Kamloops, British Columbia, Canada; Department of Medicine, Dalhousie University, Halifax, Nova Scotia, Canada; Interdisciplinary School of Health Sciences, Faculty of Health Sciences, University of Ottawa, Ottawa, Ontario, Canada; Department of Psychology, University of Toronto, Toronto, Ontario, Canada; Division of Geriatric Medicine, Dalhousie University, Halifax, Nova Scotia, Canada; Department of Psychology & Health Studies, University of Saskatchewan, Saskatoon, Saskatchewan, Canada; Department of Computer Science, Thompson Rivers University, Kamloops, British Columbia, Canada; Department of Computer Science, University of Saskatchewan, Saskatoon, Saskatchewan, Canada

**Keywords:** ageism, stigma, dementia, election, politics

## Abstract

**Background and Objectives:**

When the 2024 U.S. Presidential election was announced, Joe Biden and Donald Trump were two of the oldest candidates in election history. This circumstance created sentiments of ageist political discourse and arguments for presidential age limits. Despite clear ageist discourse during the U.S. election, there is a notable lack of research examining this issue. This study used posts from X (formerly Twitter) to understand ageism on social media during the 2024 U.S. Presidential Election, particularly focusing on the campaign period when the race was between Biden and Trump.

**Research Design and Methods:**

Posts were collected from X during the American presidential election campaign from February 11–25, 2024. After filtering out non-English, incomplete, and unrelated posts, 1,254 relevant posts were coded line-by-line and then thematically analyzed. Rigor was established by using multiple strategies ranging from a strong audit trail to using interrater reliability during thematic analysis.

**Results:**

Four main themes were identified: (1) old age as an inherent weakness: “they’re both too old,” (2) dementia-related stigma, (3) dehumanization of older adults: “ancient fossils are running for office,” and (4) fear of perceived incompetence.

**Discussion and Implications:**

Our study’s findings shed light on how ageist discourse on social media threatens the credibility of older political leaders by shifting the focus from policies to stereotypical age-based attacks. Further research is needed to examine the impact of ageist discourse on electoral campaigns.

## Background and objectives

Ageism is a critical societal issue. Ageism is defined as discrimination, prejudice, and stereotypes related to an individual’s perceived age (Butler, 1969). According to the World Health Organization (2021), an estimated one in two people are ageist against older adults. Issues of ageism have been documented by numerous literature reviews (Allen et al., 2022a; Chang et al., 2020; Hu et al., 2021) and shown to impact all aspects of society from culture to politics (Allen et al., 2022a; Chasteen et al., 2023; Gans et al., 2023; Kang & Kim, 2022). Older adults who experience ageism are more likely to experience adverse health effects, including reduced mental and physical health, social isolation, and early mortality (Butler, 1969; Chang et al., 2020; Hu et al., 2021). Given that ageism is one of the most socially acceptable forms of discrimination (Allen et al., 2022b; Angus & Reeve, 2006; Wilson et al., 2019) and with the population aging (World Health Organization, 2024), the need to understand ageism has become increasingly urgent.

Recently, ageism was prominent during the 2024 U.S. Presidential Election, as the candidates’ ages became the focus of the campaigns which fueled stereotypes about older adults (­Paniagua, 2023; Reilly, 2025). More specifically, when the 2024 U.S. Presidential Election was announced, Joe Biden and Donald Trump were two of the oldest political candidates in American election history, contributing to ageist political discourse and arguments for presidential age limits (The Lancet Healthy Longevity, 2024). Ageism is often described as the last “ism,” or form of discrimination, that remains unchallenged and is still considered socially acceptable (Allen et al., 2022b; Angus & Reeve, 2006; Wilson et al., 2019). Despite the predominance of ageism during the U.S. election, and its significant impact on the quality of life of older adults, there is a paucity of research examining this issue, especially within the political context.

In 2024, the social media platform X (formerly Twitter) had an estimated 600 million monthly active users worldwide (eMarketer, 2022) which provides a large audience for information to spread rapidly. However, social media may amplify issues of ageism. Studying ageism on social media during the U.S. Presidential Election is especially relevant because it can help to uncover how age-based stigma and discrimination are created and spread. Using data from X, this study examines ageist discourse on social media during the 2024 U.S. Presidential Election, particularly focusing on the period when the race was between Biden and Trump. Our study’s findings may provide valuable insight for policymakers, health professionals, and community leaders working to address issues of ageism against older adults.

## Research design and methods

### Ethics

Our study was guided by ethical guidelines developed for social media research (Panel of Research Ethics, 2022; Townsend & Wallace, 2016). It is important to note that ethical recommendations for social media research are different from traditional research because it does not involve the participation of human subjects (Townsend & Wallace, 2016). Specifically, our research focused exclusively on secondary information that was publicly available on X. Given that there is no required login on X and the information was publicly available information, informed consent was not required. However, to support the confidentiality of the users, we removed the usernames during the filtering and cleaning process of the posts.

### Data collection and sample

We purchased a developer account to access the Application Programming Interface (API) of the social media platform X. The API enables different pieces of software to communicate with each other. Then, we employed the Tweepy library using X API to collect tweets posted between February 11, 2024, and February 25, 2024. Tweepy, a Python-based tool, facilitated access to X data streams, enabling the systematic extraction of publicly available tweets during the timeframe. A Python-based tool is an application that is developed using the Python programming language.

Our search terms focused on examining ageism during the U.S. election (refer to Table 1). Our ageist search terms were created by drawing on a previous study on vaccine-related ageism on social media during the COVID-19 pandemic (Bacsu et al., 2024). For example, our search terms incorporated a glossary of ageist-language published in the American Association of Retired Persons (Duarte & Albo, 2018), and a list of negative terminology often used on social media to describe older adults (Centre for Ageing Better, 2021).

**Table 1.** Search terms.

**Concept**	Keyword
**Ageism**	“old hag” OR “old codger” OR “sad old” OR “bitter old” OR “old coot” OR “wee granny” OR “grumpy old” OR “boomer remover” OR “most selfish generation ever” OR “old bastard” OR “ancient” OR “oldies” OR “old timer” OR “boomer” OR “old fart” OR “codger” OR “coot” OR “geezer” OR “old grumpy” OR “grumpy old” OR “crazy old” OR “old crazy” OR “old sad” OR “old bitter” OR “old biddy” OR “old bag” OR “old bat” OR “old fogey” OR “senile” OR “demented” OR “dementia” OR “Alzheimer”
**Election**	Election
**United States**	“American” OR “America” OR “United States” OR “US” OR “USA”

**Figure 1.**
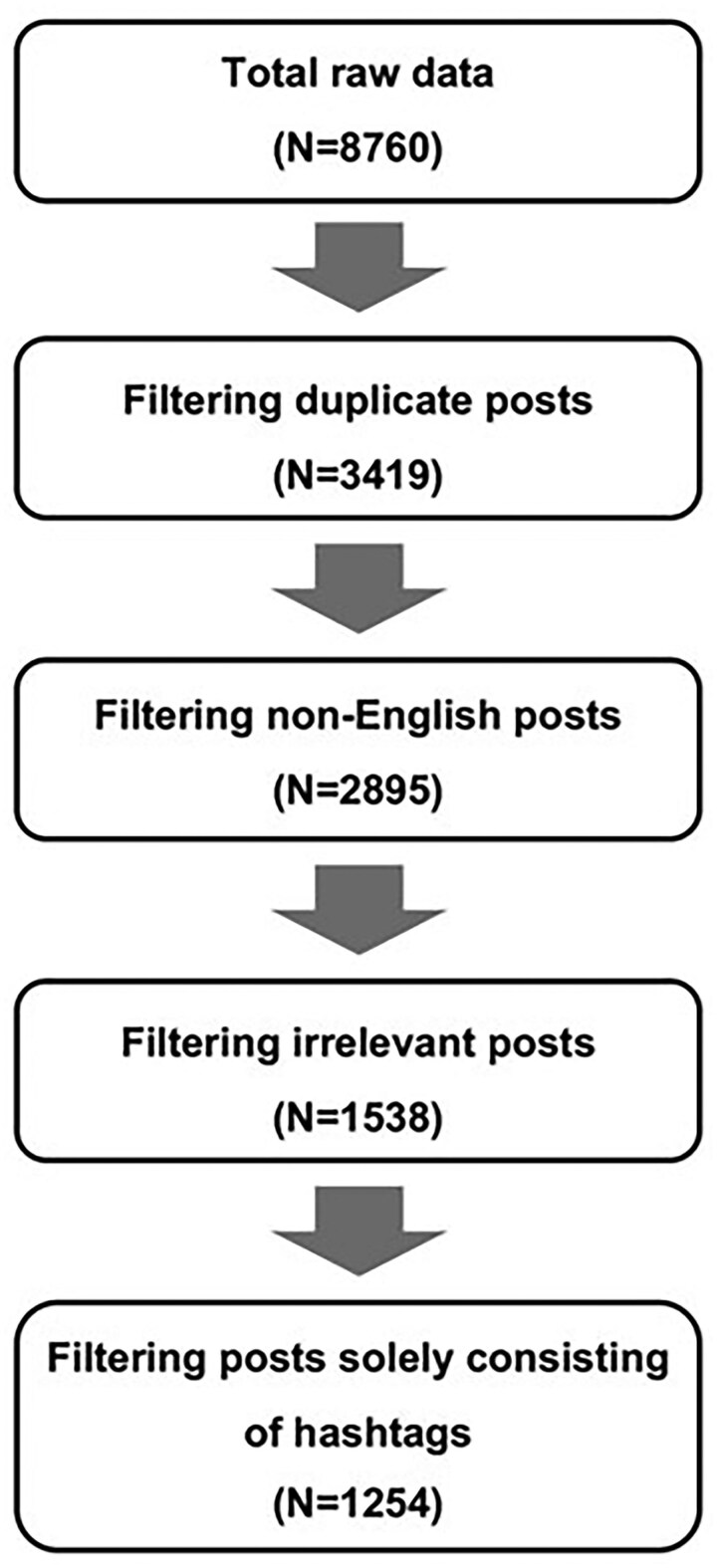
X filtering process of the tweets.

Our search identified a total of 8,760 posts. We filtered the tweets to exclude non-English posts, duplicate posts, reply posts, and posts consisting solely of hashtags. For example, reply posts were considered irrelevant because they often lacked important context, making it challenging to understand their meaning correctly. Without information of the original post, the use of reply posts risked misrepresenting and biasing the data. The filtering process is illustrated in Figure 1. The remaining 1,254 posts were analyzed using thematic analysis.

### Data analysis

Braun and Clarke’s (2006, 2021) thematic analysis framework was utilized to analyze the data. First, a cohort of the research team worked together to create a codebook. The codebook was created by reading and re-reading 250 tweets line-by-line to develop an initial list of codes. Next, the team performed practice coding exercises to ensure the codebook captured the data throughout the tweets. Throughout this process the codebook was revised and refined until it consisted of seven codes: (1) questioning cognition, (2) name-calling that excluded the term “dementia,” (3) references to “weak,” “weakness,” or “fragile,” (4) dementia-related name calling, myths, and false beliefs (5) political “puppet” labels, (6) old age is bad, and (7) ageist metaphors. Code definitions and examples were added to the codebook to ensure clarity in each of the codes.

**Figure 2.**
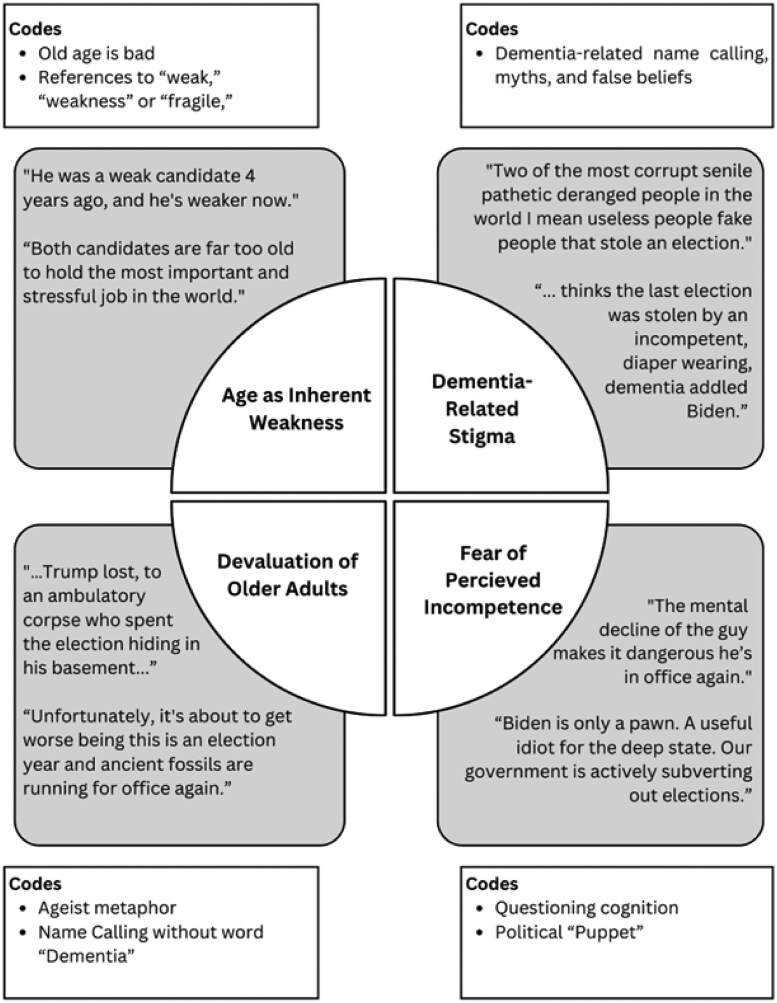
Thematic map.

Once the codebook was completed, practice coding exercises were completed with the full team to support intercoder reliability. For example, team members independently coded 100 tweets and were given detailed answer keys to compare with their coding. Additionally, team coding exercises were held with the full team, which coded 50 tweets collaboratively as a group to ensure clarity of the coding process. Once team members felt comfortable with coding, each member was assigned tweets to individually code, and coding pairs were assigned to each member to compare coding findings. The usage of coding pairs ensured intercoder reliability and high-quality interpretation of the tweets. Then, a cohort of the researchers worked to develop a theme team to identify the themes. More specifically, the theme team met and created a thematic map by grouping the codes together to make overarching themes. Our process of conceptualizing and sorting the codes into themes is illustrated in Figure 2. Themes were then reviewed and refined by the entire team to ensure consistency and clarity.

### Rigor

Rigor was established by using multiple strategies ranging from a strong audit trail to using interrater reliability during thematic analysis. For example, credibility and trustworthiness were supported by holding peer debriefing during the coding process, organizing team practice coding sessions, and hosting regular team meetings to resolve any challenges or disagreements during the coding process. Additionally, we used intercoder reliability by having pairs of coders independently code and cross-check each other’s coding. Our intercoder reliability score was analyzed by calculating the percent of agreement between the different coding partners (two coders independently coding each tweet) and then taking each of the pairs’ percentage of agreement numbers to calculate the group average, which was 85% agreement (Table 2). Confirmability was accomplished through researcher triangulation, which drew on our team’s interdisciplinary expertise such as nursing, nutrition, psychology, public health, gerontology, computer science, and geriatrics. Our team also created a comprehensive audit trail (by documenting code definitions, keywords, and X tweet examples) to outline how our research was conducted to enable other researchers to replicate our study. Moreover, our comprehensive audit trail also included keeping a detailed record of the research methods and tools such as the search terms (Table 1), the X filtering process (Figure 1), and creating a thematic map (Figure 2) to document our team’s grouping of the codes to generate the overarching themes. Collectively, these strategies ensured that findings were data-driven and systematically derived, meeting the rigor standards for qualitative research.

**Table 2.** Intercoder reliability.

**Coding partner**	Coding partner	Percentage of agreement
**Raymond Spiteri**	Ali Akbar Jamali	85.0
**Shoshana Green**	Megan O’Connell	93.0
**Sarah Fraser**	Megan Funk	88.0
**Shirin Vellani**	Alison Chasteen	89.0
**Melissa Andrew**	Jasmine Mah	77.0
**Melba D’Souza**	Juanita Bacsu	82.0
**Florriann Fehr**	Alixe Ménard	80.0
**Intercoder reliability average**		85.0

## Results

Based on our thematic analysis, four main themes were identified including: (1) old age as an inherent weakness: “they’re both too old,” (2) dementia-related stigma, (3) dehumanization of older adults: “ancient fossils are running for office,” and (4) fear of perceived incompetence.

### Age as inherent weakness: “they’re both too old”

Age was often discussed as being an inherent weakness of President Biden and former President Trump. More specifically, the candidates were often described in terms of being fragile, vulnerable, feeble, weak, and not strong enough to be the president of the United States. These discussions often depicted old age as hindering the candidates’ ability to continue to govern over time. This is consistent with broader societal ageism and negative stereotypes that often portray older adults in terms of being weak and fragile (Levy et al., 2014).

You won’t be voting for Biden… old Sniffy is too weak and too far into his dotage to hang around for another election. My guess is that they pull him out in July if he doesn’t do something that forces them to move sooner

In the November election Trump will be 78 yrs old. Biden will be 81 yrs old. Only 3 yrs apart, but both are far too old to hold the most important and stressful job in the world…

In the November Election. Trump will be 78. Biden will be 81. They’re both too old.

I agree with you. The age issue is a both sides issue, and in a normal universe would be one of significance in the election. Biden’s age is a concern, as is Trumps, but Trump’s capacity is of far greater concern…

These perceived age-related weaknesses of the candidates were often discussed in relation to international politics and relations with Russia. More specifically, posts often made reference to Russia’s desire for the United States to have a weak leader.

Vladimir Putin endorsed Biden, he knows how weak and fragile he is and would have no problem dealing with the old fart. Did Russia help Biden win or steal the election too?

Whilst Biden is better than Trump. He is not strong enough to win the next elections and is too old. (Sad but true) the chaos we see in the world is because it senses US weakness.

### Dementia-related stigma

Accusations of dementia and cognitive impairment as political weapons were at the core of many tweets, framing electoral candidates as inherently disqualifying. Several tweets shared descriptions of the political candidate’s name attached to the word dementia or senile, such as “Dementia Joe” or “Senile Biden.” There were sometimes action items added to the name-calling related to stereotypes of behaviors that would occur related to having dementia. These stigmatized tweets are illustrated in the examples below:

Demented Biden won’t make it to the election even with pumping him with tons of pills…

Donald Trump lost the 2020 election to a senile, incompetent geezer.

The tweets frequently employed terms like senile, demented, and dementia patient as pejorative labels, reinforcing negative stereotypes associated with cognitive impairment. Many posts illustrated the interplay between ageism and ableism by reinforcing the stereotype that aging inevitably leads to disability, specifically cognitive impairment.

No, Trump definitely has dementia. He’s not as far gone as Biden but it’s pretty close. This election is a joke.

You’re projecting again. Demtards are shitting their pants wondering if Dementia Joe will start drooling before the election.

The repeated conflation of being older with having dementia reflected a dual prejudice that devalues both age and ability. The use of terms like “dementia-riddled,” “not of sound mind,” or “failed memory” reflected ableist attitudes that equated cognitive impairments with incompetence, invalidity, or unworthiness.

### Devaluation of older adults: “ancient fossils are running for office”

The theme of devaluation of older adults consists of tweets that used ageist metaphors and dehumanizing language to devalue older adult candidates in the presidential election. The use of ageist language in political discourse can shape public perception of older adults’ capabilities and their rightness for leadership roles. For example, posts described the candidates using negative and degrading words that were dehumanizing. These posts often included metaphors and analogies such as describing the candidate to be like a human cucumber, donkey, ancient creature, old fossil, or inanimate object with an ageist metaphor. Some examples of the tweets are illustrated below:

Unfortunately, it’s about to get worse being this is an election year and ancient fossils are running for office again.

Donald Trump is so weak that he lost an election to a human cucumber while he (Trump) was the incumbent.

Posts also included stereotypes that depicted the candidates as being near death or past death. For example, posts highlighted the mortality of the candidates by using phrases such as an “ambulatory corpse,” “zombie,” “walking corpse,” and “dying banana.” This focus on the mortality of the candidates is depicted in the following tweets:

…Trump lost, to an ambulatory corpse who spent the election hiding in his basement…

Fat chance I'm voting for the senile dishonest corrupt ­ILLEGITIMATE President Biden! Only fools will be voting for that zombie…

The walking corpse President that intends on using illegal invade votes to fix all future elections…

American dream is to actually leave #america and live somewhere else. Literally. why is this old fossil alive? look at that face, its like a dying banana.

### Fear of perceived incompetence

The theme of fear of perceived incompetence was at the core of many ageist tweets during the U.S. election. Tweets discussed the candidates’ cognition and mental state as being a threat or dangerous to American society. For example, an X user stated, “The mental decline of the guy makes it dangerous he’s in office once again.” This fear was often described in discussions related to the economy, immigration, corruption, and war. The fear of perceived incompetence is illustrated in the following tweets:

The fear is palpable, too… Hard to tell it’s an election year as well they are banking on a corrupt, pedophile, dementia-riddled, economy destroying, war-mongering cadaver lol.

…”World will self destruct into civil and world war with Dementia Joes re election……America knows it.

Biden is the WORST president in this nation’s history…how’s he going to win an election with current immigration, inflation, and the world at war and not to mention his mental state?

This theme often intersected with dementia-related ridicule, creating a complicated narrative that questioned the candidates’ cognitive abilities and their autonomy as leaders. For example, phrases such as “puppet,” and “pawn,” were used to argue that older political leaders can be easily manipulated. More specifically, many of these posts used dementia-related insults to insinuate that the candidates were mere puppets because they did not have the cognitive ability (“dementia patient,” “brain dead,” and “mentally incompetent”) to govern on their own. Examples of these tweets are provided below:

A robot would be better than the Dementia patient who campaigned from his basement, an installed puppet in the White House that enemies of America have had control of since they highjacked our elections.fear

China hacked into the 2020 election to flip votes to help steal the election for brain dead Biden the compromised, corrupt, mentally incompetent, socialist, puppet who they own through Blackmail.

Posts also argued that cognition tests should be required for candidates to run in the presidential election. These arguments are expressed in the following posts:

Should the RNC force Trump to take a cognitive test before the general election? It seems like a necessary thing to do, because of his advanced age, & his crazy incoherent rants where he mixes up dates & names.

Biden won’t even take a cognition test BEFORE the election. It’s one thing a president developing mental disease after election like Reagan, but before the election? Nuclear codes in his hands?

## Discussion and implications

Our study explored social media discourse on ageism during the 2024 U.S. Presidential Election. Understanding ageism during the election is critical to analyzing how ageist attitudes manifest and spread. Additionally, examining the normalization of political ageism on social media may impact how older adults self-perceive old age. Our research identified four main themes ranging from the perception of old age as an inherent weakness to dementia-related stigma. Our findings shed light on the pervasive and damaging nature of ageist discourse on social media.

Although social media can be used to share political platforms and campaign information, it can also propagate ageist political discourse. For example, we found that ageist discourse was highly prevalent on social media during the election, which creates concerns about the impact of ageism and its impending consequences beyond politics to the broader societal level. Research has shown that ageism significantly increases the economic costs of health. For example, research indicates that ageism related to age-related stereotypes and poor self-perceptions of aging exacerbates older adults’ health conditions, contributing to an annual cost of US$63 billion (Levy et al., 2020).

Political ageism not only marginalizes older candidates, but it also reinforces societal stereotypes against older adults more generally. This is particularly troubling because ageism is strongly connected to adverse health outcomes. Studies show that older adults who are subjected to ageism face a higher risk of reduced physical health, poorer mental well-being, social isolation, and premature death (Butler, 1969; Chang et al., 2020; Hu et al., 2021). Despite these consequences, there remains a paucity of research focused on counteracting the negative implications of ageism, especially on digital platforms.

Findings from our study shed light on the detrimental impact of ageism during the election campaign. For example, ageist language and metaphors were often used to devalue and discredit prominent political candidates in the campaign. Research suggests that the strong prevalence of ageist political discourse on social media may contribute to a self-perpetuating cycle of negative attitudes toward older adults in leadership positions (Vauclair et al., 2015). Accordingly, it is important to recognize the broader implications of ageism beyond political discourse on social media to the societal level.

Findings from our study suggest that ageist discourse and fear of perceived incompetence may influence public opinion during the election. For example, concerns surrounding the cognitive decline of the older candidates led to calls for cognitive testing (Lamont et al., 2015). The fear of perceived incompetence of older adults, as reflected in the posts, demonstrates the complex interaction of ageism and fear-mongering to undermine older adults’ intellect and leadership capacity. Research shows that ageist stereotypes can impact older adults’ performance on cognitive and physical tasks (Murman, 2015). This phenomenon, known as the age-based stereotype threat, can lead to underperformance when older individuals are exposed to negative age-related stereotypes (Klimova & ­Dostalova, 2020; Mariano et al., 2020). The public discourse surrounding the cognitive abilities of older politicians may unconsciously contribute to this effect (Fisher et al., 2021; von Humboldt et al., 2024). Accordingly, further research is required to examine the impact of ageism on political campaigns.

Our study also identified that many posts contained terms such as “puppets,” “traitors,” “enemies of America,” and accusations of election treason that reflected an adversarial view of the electoral system. It is important to note that these posts may be more part of political strategy or concerns related to issues of corruption rather than ageism. However, when such discourse intersects with strong ageist rhetoric, especially issues of dementia-related stigma (“brain dead,” and “dementia patient”), it is critical to address the detrimental nature of ageism. The dementia-related framing and intersection with ageist statements suggests that this is not purely a matter of corruption concerns but part of a broader issue of ageist political rhetoric. Accordingly, there is a need for future research to better understand the intersection of ageism, dementia-related stigma, and political leadership in contemporary electoral discourse.

### Limitations

Although steps were taken to ensure high-quality research, it is important to recognize that our study has limitations. For example, a noteworthy limitation of our research is that it is based on social media data collected from the X platform, which may limit the generalizability of our study’s findings to the broader population. Specifically, our research removed all identifying information, and we did not collect information related to users’ socioeconomic status, geographic location, political ideology, sex and/or gender, or age. Research suggests that older adults may be marginalized in online media because they may lack the equipment (such as computers or smartphones), choose to not use it, have no or limited network connectivity, or be inexperienced in using technology (Seifert et al., 2021). Consequently, research shows that X users are typically more educated, younger, and have higher salaries than the general U.S. population (Greenwood et al., 2016).

Another limitation is that our data collection was restricted to a 2-week timeframe due to changes in X’s data access policies. For example, X has created restrictions that require payment for extended data access through their API. Based on these restrictions and the related expenses, we focused our study on a 2-week span. However, it is important to note that this short timeframe is not fully representative of the entire presidential election.

Another limitation of our study is the exclusion of images (such as photos, emojis, and GIFs) from our analysis of the posts. For example, omitting images from our dataset may limit cues that are often used to convey nuances such as sarcasm or humor. As a result, our analysis might miss certain nuances that are conveyed visually on the platform. However, it is worth noting that each of the posts was analyzed individually by two team members to support high-quality interpretations of each post.

## Conclusion

Our study examined social media discourse on ageism during the 2024 U.S. Presidential Election between candidates Joe Biden and Donald Trump. Our research identified four main themes ranging from the perception of old age as an inherent weakness to dementia-related stigma. Findings from our study shed light on how ageist discourse on social media threatens the credibility of older political leaders by shifting the focus from politics to stereotypical age-based attacks. Further research is needed to understand the impact of ageist political discourse on electoral outcomes.

## Supplementary Material

gnaf166_Supplementary_Data
