# Supplementary material for: Ageism during the 2024 U.S. Presidential Election: thematic analysis of tweets
Source: Gerontologist. 2025 Jul 26;65(10):gnaf166. doi: 10.1093/geront/gnaf166 (PMC12476580; doi:10.1093/geront/gnaf166)
Supplement: gnaf166_Supplementary_Data [file gnaf166_supplementary_data.zip › Bacsu et al. Suppl.docx]

Supplementary Material: Codebook

| **Code Numbers** | **Definition** | **Cues** | **Examples** |
| --- | --- | --- | --- |
| 1. **Missing information, irrelevant, or unclear** | Unclear or missing information (e.g., acronyms or incoherent).  Does not have intersection between ageism and election. | - Unclear/confusing - Missing information - Unknown acronyms - Irrelevant - Observations | *Since 2015,* ***Russia*** *has been working to undermine our democracy & sow chaos in elections.* |
| 1. **Questioning cognition** | Questions cognition and/or mental ability/state of a candidate, including dementia diagnosis. | - Mental state - Mental incapacity antics - Mental ability - Mental faculties - Cognition - Dementia diagnosis | *If his* ***mental faculties*** *seem up to the job. He is in his 70's after all.* |
| 1. **Name calling without** the word **“dementia”** | Name calling without the word “dementia.” | - Old fart - Old bag - Idiot - Old biddy Biden - Poor old bastard | *What needs to change beyond states like PA not violating their own constitutions to flood the zone with mail-ins for* ***old biddy Biden'*** |
| 1. Uses words “**weak,” “weakness,” or “fragile”** | Makes reference to being weak, fragile, or weakness. | - Weak - Fragile - Weaknesses | *Trump is a weak coward with a very fragile ego!* |
| 1. **Dementia**-related **name calling, myths, and false beliefs** | Any dementia-related name calling (e.g., senile and dementia), myths, and false beliefs. | - **Dementia** Joe - **Senile** - Demented president - Demented - Alzheimer’s | *Election time….PLEASE...get a new playbook. This one is getting as old as this* ***DEMENTED elderly old codger*** *is.* |
| 1. **Political “puppet”-** | Notion that candidate is a puppet due to old age or inability to think on their own. | - Demented **puppet** president - Puppet-master - puppetry | *Biden didn't win an election, he was a… demented president who is a mouth piece for his* ***puppet masters-****master, OBAMA!!* |
| 1. **Old age is bad** | Implies that “**old age”** is a detriment. Focuses on age stereotypes and notion that age is a bad thing. | - **80-year**-old Biden   -I don’t care about his age that much either… | *Trump's court cases vs Biden's age issue - Will the two repeat the 2020 battle?* |
| 1. **Ageist metaphor** | Any ageist metaphors that excludes puppet. | - **Cucumber** - **Zombie** | *Fat chance I'm voting for the senile corrupt ILLEGITIMATE resident Biden! Only fools will be voting for that* ***zombie.***  *Trump lost election to human* ***cucumber…*** |
